# Molecular identification of *bla*TEM gene of extended-spectrum beta-lactamase-producing *Escherichia coli* from healthy pigs in Malang district, East Java, Indonesia

**DOI:** 10.5455/javar.2022.i613

**Published:** 2022-09-30

**Authors:** Mustofa Helmi Effendi, Erwan Budi Hartadi, Adiana Mutamsari Witaningrum, Dian Ayu Permatasari, Emmanuel Nnabuike Ugbo

**Affiliations:** 1 Department of Veterinary Public Health, Faculty of Veterinary Medicine, Universitas Airlangga, Surabaya, Indonesia; 2 Postgraduate Student of Veterinary Public Health Program, Faculty of Veterinary Medicine, Universitas Airlangga, Surabaya, Indonesia; 3 Department of Applied Microbiology, Ebonyi State University, Abakaliki, Nigeria

**Keywords:** *Bla*TEM gene, ESBL, *Escherichia coli*, pigs, public health

## Abstract

**Objective:**

The increase and prevalence of multidrug-resistant bacteria in livestock animals are serious public health concerns. This study aimed to identify the presence of the *bla*TEM gene in extended-spectrum beta-lactamase (ESBL)-producing *Escherichia coli* isolated from rectal swabs of apparently healthy pigs in Malang District, East Java, Indonesia.

**Materials and Methods:**

A total of 120 rectal swab samples were collected from the pigs. The rectal swabs were screened for the presence of *E. coli* using standard microbiological identification procedures. The Kirby–Bauer disk diffusion method identified multidrug-resistant *E. coli*. Five different classes of antibiotics were used to identify multidrug-resistant isolates, including Ciprofloxacin, Trimethoprim, Tetracycline, Streptomycin, and Aztreonam. Multidrug-resistant *E. coli* isolates were characterized for the presence of ESBL using double-disk synergy test methods. The presence of *bla*TEM genes was determined using polymerase chain reaction methods.

**Results:**

The results of this study indicated that 107 (89.2%) out of 120 samples analyzed were positive for *E. coli* isolates. A total of 32 (29.9%) *E. coli* isolates were identified to be multidrug-resistant and further subjected to molecular testing. The molecular analysis revealed (5; 15.6%) *E. coli* isolates to harbor the *bla*TEM gene.

**Conclusion:**

The results of this study revealed that pigs and products of pork origin must be considered a source of transmission of ESBL-producing *E. coli* to public health important under the food chain.

## Introduction

The cause of disease in pigs by bacterial agents is a problem that is often faced by managers of pig farms. This has led to the use of antibiotics for the prevention and treatment of the disease [1]. Irrational and inappropriate uses of antibiotics can increase the incidence of multidrug resistance (MDR) [2,3]. The irrational use of antibiotics by pig farmers contributes greatly to bacterial resistance to antimicrobial agents [4]. The harmful impact caused by bacterial resistance to antibiotics is that the treatment time for bacterial diseases becomes longer or the treatment fails. Less effective treatment impacts the length of treatment and the use of drugs that are more expensive and, of course, the costs incurred [5,6]. The use of *Escherichia coli* bacteria, other than being an indicator of the level of sanitation in livestock, also acts as a reservoir for the spread of resistance genes by transferring resistant genes to other bacteria. One of the signs and characteristics of *E. coli* that can spread resistance genes is its ability to form MDR and produce extended-spectrum beta-lactamase (ESBL), which can hydrolyze the beta-lactam ring [7].

Previous studies have shown that ESBL-producing *E. coli* have been isolated from animals, hospital environments, plants, water, and feces [5]. Several studies have also reported a high prevalence of ESBL-producing *E. coli* in farm animals [5,7]. It can transmit plasmids containing the gene encoding ESBL from the natural environment to humans or livestock [8,9]. *Escherichia* *coli producing* MDR is a serious threat to animal and human health. It also causes a disease that often occurs in pigs from birth to weaning, characterized by white to yellow diarrhea. This disease is known as colibacillosis [10]. Antibiotic-resistant *E. coli* can be spread from animals to humans through the food chain, direct contact, or the environment [11].

The occurrence of beta-lactam antibiotic resistance genes in *E. coli* isolated from animals has attracted much attention, especially in organisms with the potential to transfer resistance genes [12]. The spread of resistance genes can be mediated through horizontal genetic transfer mechanisms such as conjugation, transformation, and transduction [13]. There are three main genes encoding ESBL, namely TEM, SHV, and CTX-M, with the* bla*TEM gene being the most commonly found in community and livestock environments [14–16]. These three genes play a role in producing ESBL capable of hydrolyzing beta-lactam antibiotics. This may cause these antibiotics to become ineffective as the treatment progresses. These genes are located on bacterial plasmids that can spread easily between and within bacterial species [17,18].

This study aimed at the molecular identification of the *bla*TEM gene of ESBL-producing *E. coli* from apparently healthy pigs. This is related to biosafety and is based on cases of high resistance of *E. coli* to antimicrobial agents used in treating human diseases, which can also be sourced from animals or livestock treatments.

## Materials and Methods

### Ethical approval

Animal ethics approval was obtained via the ethical clearance commission of Universitas Airlangga, Indonesia (ethics no.: 353/HRECC/VI/2021).

### Sample collection, isolation, and identification

One hundred twenty (*n* = 120) rectal swabs were collected from three pig farms in the Malang district, East Java, Indonesia. The rectal swab samples were collected using Amies transport media (Delta lab), stored in a cool box, and transported to the laboratory at the Department of Veterinary Public Health, Faculty of Veterinary Medicine, Universitas Airlangga, for immediate analysis. The samples were cultured on eosin methylene blue agar (EMBA) media (Merck; 101,347) for 24 h at 37°C [19]. Colonies of suspected *E. coli* on EMBA media grew to a metallic green color (20). Then, the pure cultures of the suspected *E. coli* colonies were subcultured again on EMBA. The suspected *E. coli* colonies growing on EMBA media were stained using the Gram Staining Kit (HiMedia; K001–1KT) to confirm morphology and bacterial properties. Furthermore, the suspected isolates of *E. coli* were identified using the indole, methyl red, Voges–Proskauer, in citrate biochemical test. *Escherichia coli* showed positive indole results and motility on sulfide indole motility media (Merck; 105,470). The identified *E. coli* isolates were subjected to MDR, ESBL, and polymerase chain reaction (PCR) screening tests [20,21].

### Antibiotic susceptibility testing and phenotypic test for ESBL detection

*Escherichia coli* isolates isolated from pig rectal swabs were tested for MDR using the Kirby–Bauer diffusion method. The Mueller Hinton agar medium (Merck; 105,437) was prepared according to the manufacturer’s instructions. An overnight incubated (37°C) pure culture of *E. coli* isolates in nutrient broth was adjusted to 0.5 McFarland turbidity. The bacterial isolates were inoculated on the plates. Different classes of antibiotics (Ciprofloxacin 5 µg, Trimethoprim 5 µg, Tetracycline 30 µg, Streptomycin 10 µg, and Aztreonam 30 µg) (Oxoid CT0264B) were placed on the surface of the Mueller Hinton agar and incubated at 37°C for 18–24 h [22,23]. The results of the inhibition zone diameter were interpreted according to the Clinical Laboratory Standards Institute [24]. The results were recorded in qualitative categories with sensitive, intermediate, and resistant ratings [22,23]. Isolates that showed reduced sensitivity to two or more different classes of antibiotics were recorded as MDR. Phenotypical detection of ESBL-producing *E. coli* was carried out using double-disk synergy according to a method previously described [25].

### Molecular identification of the blaTEM gene by PCR

The *E. coli* identified as MDR and ESBL producers phenotypically were further subjected to genotype analysis for the presence of the *bla*TEM gene using the PCR molecular identification method. Bacterial DNA was isolated using the QIAamp^®^ DNA Mini Kit (QIAGEN, Germany) according to the methods described previously [26]. The primers were F: ATA AAA TTC TTG AAG ACG AAA and R: GAC AGT TAC CAA TGC TTA ATC [26]. *Escherichia coli* ATCC 35218 was used as the ESBL positive control standard, and *E. coli* ATCC 25922 as the ESBL negative control standard [26]. PCR results were visualized by electrophoresis using a 2% agarose gel (Invitrogen, USA) [27,28].

## Results

**Figure 1.**
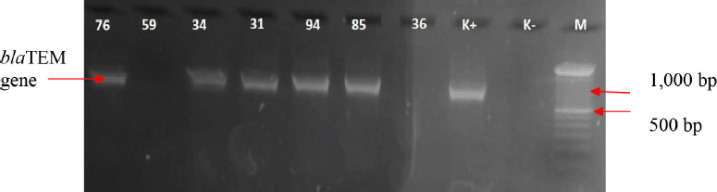
Molecular identification of the *bla*TEM gene using PCR genotyping (PCR product for *bla*TEM gene = 1,080 bp). *M* = marker 100 bp; K+ = control positive; K− = control negative; five samples of MDR cases were positive for *bla*TEM gene.

One hundred twenty rectal swab samples were collected from three farms (40 each) (Asia, Nyomo, and Krisna farms). A total of 107 (89.2%) *E. coli* samples were identified from all the farms; Asia (40/40), Nyomo (40/40), and Krisna (27/40) were identified as positive samples. Out of the 107 *E. coli* samples identified, 32 (29.9%) were confirmed MDR; Asia farm had 8/40; Nyomo farm harbored 12/40; and Krisna Farm had 12/27 (Table 1). Among the 32 MDR *E. coli* isolates isolated from the 3 farms, 5 (15.6%) ESBL-producing *E. coli* isolates were discovered (Asia–2, Nyomo Farm–1, and Krisna Farm–2) to harbor the *bla*TEM gene using the PCR molecular method (Fig. 1). Notably, most of the *E. coli* isolates that were resistant to Aztreonam (Presumptive ESBL test) were found to harbor the ESBL *bla*TEM gene.

## Discussion

This study has shown the distribution of MDR and ESBL-producing *E. coli* in three pig farms in the Malang District. Asia farm had 2 ESBL-positive samples out of 40 *E. coli* samples; Nyomo farm had 1 ESBL-positive sample out of 40 *E. coli* samples, and Krisna farm had 2 ESBL-positive samples out of 27 *E. coli* samples. Several previous studies have found the presence of *E. coli* isolates from pig farms inappropriate (Table 1) [29–31]. The number of ESBL-producing *E. coli* found in animals such as pigs, cattle, dogs, and poultry has proven that many gene variants were observed [3,32–35]. This study discovered an ESBL-producing *E. coli* with a gene encoded to be *bla*TEM. However, 15.6% (5/32) of the ESBL-producing *E. coli* isolates harbored the *bla*TEM gene out of the 32 MDR isolates. The *bla*TEM gene encoding ESBL is most often found in *E. coli* [36,37]. The molecular identification confirms a visualization of the *bla*TEM gene fragment band (Fig. 1). The *bla*TEM gene electrophoresis results in ESBL-producing *E. coli* positive isolates showed the same fragments as in positive controls with an amplicon length of 1,080 bp [38].

The presence of the* bla*TEM gene as an ESBL encoding in *E. coli* bacteria indicates that there has been a spread of bacteria that produce ESBL enzymes. These results confirm that the presence of the* bla*TEM gene may allow the spread of the resistant gene to other bacteria. Of the 32 MDR *E. coli* isolates, only 15.6% were positive for the* bla*TEM gene. The remaining inability of other MDR-producing *E. coli* to have no* bla*TEM gene found could be due to the isolate being produced by other ESBL genes other than our preferred or interesting gene. It is also possible that the total sample had other ESBL genes not examined in this study. ESBL has several classes and each class has several genes [39]. The findings of this study confirm previously published findings. In addition to the* bla*TEM gene, other ESBL genes, such as* bla*CTX-M,* bla*CMY,* bla*SHV*,* and *amp*C, have been identified in bacteria associated with infection in livestock [40]. The discovery of ESBL-producing *E. coli* isolates isolated from swine rectal swabs in this study is in line with research in Mizoram, India [38]. ESBL-producing bacteria can be identified by looking for the presence of ESBL-encoding genes, such as *bla*TEM,* bla*CTX-M, and* bla*SHV genes [15,41]. Studies from other regions have shown that the* bla*TEM gene is the most common ESBL-encoding gene and is most commonly found in cases of ESBL-producing *E. coli* originating from livestock, especially pigs [38,41]. In various countries, the* bla*TEM gene is one of the most common ESBL-encoding genes and causes infections in humans and animals [42–44]. Therefore, the discovery of the* bla*TEM gene in this study can be a major public health threat and can be a reference in controlling the spread of the* bla*TEM gene as one of the main genes encoding for ESBL-producing *E. coli* among pig farms. This study also aligns with Mandakini et al. [38], who explained that the main ESBL gene found in *E. coli* sourced from pig farms was* bla*TEM. The incidence of this case indicates that pigs and livestock products have the potential to transmit this gene to other bacteria and other hosts through various pathways [9,45].

**Table 1.** ESBL-producing* E. coli* from healthy pigs in Malang.

Location	Sample size	Isolates of *Escherichia coli*	Total no. of MDR cases	*bla*TEM gene
Asia farm	40	40	8	2
Nyomo farm	40	40	12	1
Krisna farm	40	27	12	2
Total	120	107 (89.2)	32 (29.9)	5 (15.6)

MDR, Multidrug-resistant.

Resistance genes can be widely dispersed through horizontal gene transfer mechanisms such as conjugation, transformation, and transduction. Gene transfer mechanisms mobilize specific DNA fragments from one region to another, between plasmids, between chromosomes, and between plasmids and chromosomes. Plasmid-mediated diffusion of beta-lactamase is thought to contribute to the large spread of this enzyme type worldwide [13,46,47]. The majority of resistance genes can be spread by *E. coli* horizontally to other members of the Enterobacteriaceae family via plasmids [12]. In addition, mobile genetic elements, such as transposons, insertion sequences, and integrons, in bacteria cause the ESBL gene to be easily transferred from humans to animals. Genetic elements can also spread resistance to other bacteria in the digestive tract of animals. Bacteria that contain resistant genetic elements can then be spread from farms to the surrounding environment due to poor livestock hygiene and sanitation practices through livestock manure that contaminates the soil and water around the farm. ESBL-producing bacteria have also been detected in plants, soil, and water around agricultural, livestock, and market environments [48,49]. This proves that ESBL bacteria, besides being the cause of nosocomial infections, also cause community infections and foodborne diseases. Evidence of the presence of ESBL-encoding genes detected in isolates of animal origin can threaten the public and animal health. However, different antibiotics, such as third-generation cephalosporins and monobactams, have never been used in animals [50–52]. The impact of this condition is the limited choice of appropriate antibiotic treatment in dealing with bacterial infections because many types of antibiotics are resistant. Recent studies have suggested spreading the ESBL-encoding gene from abattoir pigs [45]. Because of this, good cage management and sanitation practices, as well as how animals are killed and distributed, need to be improved so that consumers do not get diseases from animals [53,54].

## Conclusion

The current study has shown that despite the average rate of MDR (29.9%) and low rate of ESBL-producing *E. coli* that harbored the *bla*TEM gene (15.6%) observed, apparently healthy livestock animals such as pigs can harbor antibiotic-resistant bacteria in their rectum, which can also be seen in their intestines. In livestock farming, animals (pigs) are often treated with antimicrobial agents for bacterial infections; this encourages pressure that favors resistant bacteria that carry genes such as *bla*TEM, *bla*CTX, *bla*SHV, and *amp*C. The presence of the gene encoding ESBL in bacteria has the potential to spread resistance genes to other bacteria in the digestive tract of pigs, pig farming environments, and pig slaughterhouses. So, more needs to be done to show how important it is to manage housing and keep it clean, as well as how to slaughter pigs and distribute them.

## List of abbreviations

EMBA: eosin methylene blue agar; ESBL: extended-spectrum beta-lactamase; MDR: multidrug-resistance; PCR: polymerase chain reaction.
